# A possible role for inflammation in mediating apoptosis of oligodendrocytes as induced by the Lyme disease spirochete *Borrelia burgdorferi*

**DOI:** 10.1186/1742-2094-9-72

**Published:** 2012-04-23

**Authors:** Geeta Ramesh, Shemi Benge, Bapi Pahar, Mario T Philipp

**Affiliations:** 1 Division of Bacteriology and Parasitology, Tulane National Primate Research Center, Covington, LA, USA; 2 School of Science and Engineering, Tulane University, New Orleans, LA, USA; 3 Division of Comparative Pathology, Tulane National Primate Research Center, Covington, LA, USA

**Keywords:** Lyme neuroborreliosis, *Borrelia burgdorferi*, Oligodendrocytes, CCL2/MCP-1, IL-6, IL-8, Apoptosis, Active caspase-3, Dexamethasone

## Abstract

**Background:**

Inflammation caused by the Lyme disease spirochete *B. burgdorferi* is an important factor in the pathogenesis of Lyme neuroborreliosis. Our central hypothesis is that *B. burgdorferi* can cause disease via the induction of inflammatory mediators such as cytokines and chemokines in glial and neuronal cells. Earlier we demonstrated that interaction of *B. burgdorferi* with brain parenchyma induces inflammatory mediators in glial cells as well as glial (oligodendrocyte) and neuronal apoptosis using *ex vivo* and *in vivo* models of experimentation.

**Methods:**

In this study we evaluated the ability of live *B. burgdorferi* to elicit inflammation *in vitro* in differentiated human MO3.13 oligodendrocytes and in differentiated primary human oligodendrocytes, by measuring the concentration of immune mediators in culture supernatants using Multiplex ELISA assays. Concomitant apoptosis was quantified in these cultures by the *in situ* terminal deoxynucleotidyl transferase mediated UTP nick end labeling (TUNEL) assay and by quantifying active caspase-3 by flow cytometry. The above phenomena were also evaluated after 48 h of stimulation with *B. burgdorferi* in the presence and absence of various concentrations of the anti-inflammatory drug dexamethasone.

**Results:**

*B. burgdorferi* induced enhanced levels of the cytokine IL-6 and the chemokines IL-8 and CCL2 in MO3.13 cells as compared to basal levels, and IL-8 and CCL2 in primary human oligodendrocytes, in a dose-dependent manner. These cultures also showed significantly elevated levels of apoptosis when compared with medium controls. Dexamethasone reduced both the levels of immune mediators and apoptosis, also in a manner that was dose dependent.

**Conclusions:**

This finding supports our hypothesis that the inflammatory response elicited by the Lyme disease spirochete in glial cells contributes to neural cell damage. As oligodendrocytes are vital for the functioning and survival of neurons, the inflammation and subsequent apoptosis of oligodendrocytes induced by *B. burgdorferi* could contribute to the pathogenesis of Lyme neuroborreliosis.

## Background

Lyme neuroborreliosis (LNB) in the US is manifest in 10% to 15% of patients diagnosed with Lyme disease [1,2]. In addition to the classical neurological triad of meningitis, cranial neuritis, and radiculitis, LNB may also manifest, albeit more rarely, as encephalopathy, encephalomyelitis [3,4], and cerebellitis [5]. Acute transverse myelitis, caused by inflammatory processes of the spinal cord resulting in axonal demyelination, has also been reported in LNB patients [6-9]. In the peripheral nervous system (PNS), Lyme disease appears as neuritis with patchy multifocal axonal degeneration associated with epineural perivascular inflammation [10,11].

LNB patients may experience a wide array of neurological and neuropsychiatric symptoms as a result of white matter inflammation that results in a subacute multiple sclerosis (MS)-like manifestation [12,13]. Brain magnetic resonance imaging (MRI) of LNB patients that was suggestive of a demyelinating disease, with MS-like symptoms that responded well to antibiotic therapy, has been reported [14,15]. It has been hypothesized that *B. burgdorferi* may exacerbate MS or be a trigger for an MS-like inflammatory demyelinating disease of the central nervous system (CNS) by activating myelin-specific T cells via molecular mimicry [16,17], or by bystander activation via inflammatory cytokines [16].

Encephalitis associated with LNB involves white matter more often than gray matter [4,18,19]. Inflammatory lesions in the brain and spinal cord show multifocal encephalitis with large areas of demyelination in perivascular white matter commonly associated with the presence of *B. burgdorferi* DNA [6,20-22]. Astroglial and neuronal proteins, anti-myelin antibodies and cells secreting antibodies to myelin basic protein have been detected in the cerebrospinal fluid (CSF) of patients with LNB, indicating possible glial and neuronal damage in the CNS parenchyma [23-25]. There is evidence that *B. burgdorferi* spirochetes can adhere to neurons, CNS glia, and Schwann cells from studies in neuronal and glial cell lines and primary rat brain cultures [26], and that *B. burgdorferi* can adhere to and perhaps invade human neuroglial and cortical neuronal cells [27]. Adhesion was found to be associated with galactocerebroside, a glycolipid component of myelin, and oligodendrocytes in primary brain cultures were shown to be damaged, by scanning electron microscopy [26,28,29]. Cells that secrete antibodies to myelin basic protein have been found in CSF of patients with LNB, suggesting damage to oligodendrocytes possibly as a result of demyelination [24].

Cytokines and chemokines are key immune mediators that play an important role in promoting CNS injury in various kinds of inflammatory neurodegenerative diseases [30-34]. Various inflammatory cytokines and chemokines have been reported in the CSF of patients with LNB [35-38].

We hypothesize that *B. burgdorferi* can cause disease via the induction of inflammatory mediators such as cytokines and chemokines in glial and neuronal cells. Earlier we demonstrated that interaction of *B. burgdorferi* with brain parenchyma induces inflammatory mediators in glial cells as well as glial (oligodendrocyte) and neuronal apoptosis [39]. Further, we found that a similar inflammatory response occurs *in vivo,* as demonstrated in rhesus monkeys inoculated intrathecally with live *B. burgdorferi.* This resulted in elevation of IL-6, IL-8, CCL2, and CXCL13 in the CSF within 1 week post infection, accompanied with histopathological changes consistent with acute neurological Lyme disease such as leptomeningitis and radiculitis, as well as satellite glial cell and neuronal apoptosis in the dorsal root ganglia [40].

Here we assessed the ability of live *B. burgdorferi* to elicit inflammatory mediators in cultures of differentiated human MO3.13 oligodendrocytes [41], and primary cultures of differentiated human oligodendrocyte precursor cells (HOPC). Further, we examined the ability of live *B. burgdorferi* to induce apoptosis of oligodendrocytes, and quantified apoptosis in the above cultures by the *in situ* TUNEL assay, and by measuring activated caspase-3 by flow cytometry. The role of inflammation in mediating apoptosis of oligodendrocytes, as induced by *B. burgdorferi* was studied by evaluating the above phenomena after 48 h of stimulation with *B. burgdorferi* in the presence and absence of various concentrations of the anti-inflammatory drug dexamethasone, a glucocorticoid used in the treatment of immune-mediated inflammatory diseases [42].

## Methods

### Maintenance and differentiation of MO3.13 cultures

The human oligodendrocyte cell line MO3.13 was obtained from CELLutions Biosystems Inc. (Burlington, Ontario, Canada). Cells were revived as per the manufacturer’s instructions and maintained in complete growth medium (CGM) consisting of Dulbecco’s minimal essential medium (DMEM) (high glucose) (Invitrogen, Carlsbad, CA), 10% fetal bovine serum (ThermoFisher, Waltham, MA, USA), and antibiotics, 100 units of penicillin and 100 μg of streptomycin (P/S) (Invitrogen), in a humidified incubator with an atmosphere of 5% CO_2_, set at 37°C. Cells were maintained in CGM for 3 days, after which the medium was replaced by differentiation medium (DM), consisting of DMEM, P/S, and phorbol 12-myristate 13-acetate (Sigma, St. Louis, MO, USA), at a concentration of 100 nM, and devoid of serum. Cells were cultured in DM for 4 days, after which time they were used in experiments.

MO3.13 cells were also seeded in Lab-Tek II CC^2^ chamber slides containing two wells (Nunc, Rochester, NY, USA) at a density of 0.5 x 10^4^ cells per well, and maintained in 2 mL CGM followed by DM as described above for the purpose of evaluating phenotypic markers using immunofluorescence staining and confocal microscopy, as well as for evaluation of apoptosis by the *in situ* TUNEL assay. Typically, the final cell count in chamber slides after maintenance in CGM for 3 days followed by DM for 4 days was 2.5 x 10^4^ cells per well. Cells were seeded into six-well plates at a seeding density of 2 x 10^4^ cells per well for evaluation of inflammatory mediators and for flow cytometry experiments. Typically, the final cell density after differentiation in six-well plates was 2.5 x 10^5^ cells per well. Only differentiated MO3.13 cells were used for estimation of inflammatory mediators or for the evaluation of apoptosis, described below.

### Human oligodendrocyte precursor cells (HOPC)

HOPC were cultured on poly-L-Lysine coated chamber slides containing two wells at a seeding density of 8 x 10^4^ cells per well, as recommended by the provider (ScienCell Inc., Carlsbad, CA, USA). Cells were revived by thawing cultures as per the manufacturer’s instructions and maintained in ‘precursor medium’ for 8 days, after which they were maintained in ‘differentiation medium’ for 3 days prior to commencing experiments. Both media were supplied by the manufacturer, and their composition is proprietary. The final cell count after differentiation was comparable to the initial seeding density. The HOPC differentiated into mature cells with longer cell processes, as indicated by the manufacturer. Differentiated HOPC maintained on poly-L-Lysine-coated chamber slides were used for the evaluation of both secreted immune mediators as well as apoptosis by the *in situ* TUNEL assay.

### Stimulation of differentiated MO3.13 oligodendrocytes and HOPC cultures with live *B. burgdorferi* for evaluation of immune mediators and apoptosis

*B. burgdorferi* strain B31 5A19 passage 3 was grown in Barbour-Stoenner-Kelly-H (BSK-H) medium, supplemented with 6% rabbit serum (Sigma, St. Louis, MO, USA) and antibiotics (rifampicin at 45.4 mg/mL, phosphomycin at 193 mg/mL and amphotericin at 0.25 mg/mL) to late logarithmic phase under microaerophilic conditions. Spirochetes were pelleted at 2000 x g for 30 min at RT. At the end of the run the rotor was left to coast without breaking so as to minimize damage to the live spirochetes. The differentiated MO3.13 cultures were washed in DM devoid of P/S. The *B. burgdorferi* culture was washed twice using phosphate buffered saline (PBS) pH 7.2 (Invitrogen, Grand Island, NY, USA) and resuspended in DM at a concentration so as to achieve the desired multiplicity of infection (MOI). Controls with no spirochetes were also included. Cultures were incubated for 48 h in a humidified 5% CO_2_ incubator, set at 37°C. At the 48-h time point culture supernatants were collected for evaluation of inflammatory mediators. Culture supernatants were centrifuged at 4°C at 2000 x g for 30 min to remove any suspended bacteria and the supernatant was aliquoted and stored at -80°C until used. The oligodendrocyte cultures were then fixed in 2% paraformaldehyde as described below for assessment of apoptosis. Spirochetes remained motile after 48-h incubation in MO3.13 or HOPC differentiation medium. Assessment of motility after incubation in MO3.13 differentiation medium required re-culturing spirochetes in BSK-H.

### Immunofluorescence staining and confocal microscopy

MO3.13 cells were either held in CGM for 3 days or further incubated in DM for 4 days for evaluation of phenotypic markers pre- and post-differentiation, respectively. Only differentiated HOPC cultures were used for evaluation of phenotypic markers.

Medium was removed and cells were fixed in 2% paraformaldehyde in PBS (PFA) (USB, Cleveland, OH, USA) at RT for 10 min with gentle rocking on a rocker in the dark. PFA was removed with three washes using PBS, each for 5 min at RT on the rocker. Cells were then given a post-fixation permeabilization treatment using a mixture of ethanol:acetic acid (2:1) (Sigma) for 5 min at -20°C. Cells were washed thrice with PBS as described above. The slides were then detached from the chamber by placing the chambers in 70% methanol for 10 min and following the manufacturer’s instructions (Nunc). Detached slides were transferred to slide holders containing PBS-FSG-TX-100 buffer (phosphate-buffered saline pH 7.4 containing 0.2% fish skin gelatin (Sigma), and 0.02% Triton X-100 (MP Biomedicals, Solon, OH, USA), and 0.02% sodium azide (Sigma), and held in this buffer for 15 min with gentle rocking at RT for permeabilization, followed by a rinse with PBS-FSG (phosphate-buffered saline containing 0.2% fish skin gelatin and 0.02% sodium azide). Slides were then blocked in a buffer consisting of PBS containing 10% normal goat serum (Invitrogen) and 0.02% sodium azide (NGS) for 1 h in a humidified chamber at RT, followed by incubation with respective primary antibodies; rabbit polyclonal anti-human myelin basic protein (MBP) Clone AB 980 at 1:100 (Millipore, Billerica, MA, USA), or mouse monoclonal IgG1 anti-human glial fibrillary acidic protein (GFAP), Clone G-A-5 at 1:200 (Sigma). Relevant isotype controls (Sigma) at the same concentrations as their respective primary antibodies were also included.

All primary antibodies at the appropriate concentrations were left on the slides for 1 h at RT, in a humidifying box. The slides were then rinsed with PBS-FSG-TX-100 buffer and then held in this buffer for 5 min, followed by a rinse with PBS-FSG buffer. The relevant secondary antibodies, either goat anti-mouse or goat anti-rabbit (Invitrogen) at a dilution of 1:1000 in NGS, were applied to the slides and left in the humidified dark slide-box at RT for 30 to 45 min. Secondary antibodies were conjugated to one of the Alexa fluorochromes- Alexa 488 (green) or 568 (red). Slides were washed and rinsed as described above and then incubated with a nuclear stain TOPRO-3 (Invitrogen) at 1:1000 in NGS for 15 min. After a final wash in PBS-FSG-TX-100 buffer followed by a rinse in PBS-FSG, slides were mounted in anti-quenching medium (Sigma). The stained and mounted slides were stored in the dark at 4°C until they were viewed under a confocal microscope. MO3.13 cultures were evaluated for the expression of MBP as well as GFAP, while HOPC cells were only stained for the evaluation of MBP expression. Slides with MO3.13 and HOPC oligodendrocytes were also stained with isotype controls for the primary antibodies at protein concentrations used for the respective primary antibodies.

Confocal microscopy was performed using a Leica TCS SP2 confocal microscope equipped with three lasers (Leica Microsystems, Exton, PA, USA). Images of individual channels were merged to obtain images containing all channels. Photoshop CS3 (Adobe systems Inc., San Jose, CA, USA) was used to assign colors to each fluorochrome.

### Evaluation of immune mediators from culture supernatants

The concentrations of cytokines and chemokines present in the culture supernatants were quantified using the Human 14-plex Cytokine-Chemokine Array kit (Millipore), following the manufacturer’s instructions. The analytes detected by this panel are: Hu IL-1β, Hu IL-2, Hu IL-4, Hu IL-5, Hu IL-6, Hu IL-7, Hu IL-8, Hu IL-10, Hu IL-12 (p70), Hu IL-13, Hu GMCSF, Hu IFN-γ, Hu CCL2, and Hu TNF-α. The multiplex plate was read using a Bio-Plex 200 Suspension Array Luminex System (Bio-Rad, Hercules, CA, USA).

### Evaluation of apoptosis by *in situ* TUNEL assay

Cells contained in chamber slides were labeled for MBP by immunofluorescence staining as described above. Slides were then fixed with 2% PFA, washed three times with PBS by rinsing slides in PBS and holding them in PBS for 2 min between washes. Slides were then subjected to the TUNEL ApopTagPlus fluorescein *in situ* apoptosis assay (Chemicon, Temecula, CA, USA) as per the manufacturer’s instructions. Slides were then mounted as described above and stored at 4°C in the dark until viewed. The percentage of apoptotic oligodendrocytes from 10 fields was evaluated from each chamber area by counting the total number of MBP-positive cells (at least 500 cells) from each of the chamber areas, followed by the number of cells that showed co-localization of both the TUNEL signal and MBP expression. All counts were made by viewing slides under a fixed magnification of 63 × (corresponding to an area of 0.05 mm^2^) using the confocal microscope.

### Evaluation of the role of inflammation in mediating oligodendrocyte apoptosis using the anti-inflammatory drug dexamethasone

MO3.13 cell cultures were seeded as described above in chamber slides for evaluation of apoptosis or in six-well plates for evaluation of immune mediators, and maintained in growth and differentiation medium as described. Prior to stimulation with live *B. burgdorferi*, differentiated cultures were incubated with various concentrations of dexamethasone (water soluble), 5 μM, 15 μM, and 150 μM (Sigma) for 24 h at 37°C, after which they were washed and then incubated in fresh differentiation medium containing the respective concentrations of dexamethasone and live *B. burgdorferi* at a MOI of 10:1 at 37°C for 48 h and devoid of P/S. Similar concentrations of dexamethasone as those mentioned above have been reported to inhibit the production of CCL2 in mice microglia [43]. Dexamethasone is supplied as a water-soluble formulation consisting of dexamethasone and a carrier substance (2-hydroxypropyl)-β-cyclodextrin. The effect of the carrier alone, at the respective molar concentrations accompanying dexamethasone was assessed by incubating MO3.13 oligodendrocytes as described above in the presence and absence of *B. burgdorferi* and carrier alone at 15, 45, and 450 μM, respectively.

After 48 h, culture supernatants were collected and processed for evaluation of inflammatory mediators, and cells were fixed and evaluated for apoptosis by the *in situ* TUNEL assay as described above. Medium controls that were pretreated and then incubated with the same respective concentrations of dexamethasone but without the addition of live *B. burgdorferi* were also included. The effect of dexamethasone on differentiated HOPC was also evaluated as described above.

### Evaluation of expression of MBP and active caspase-3 by immunofluorescence staining and flow cytometry

Differentiated MO3.13 cell cultures that were maintained in six-well plates and were stimulated with *B. burgdorferi* at a MOI of 10:1 for 48 h in DM devoid of P/S were used in this experiment. Most of the medium covering the cells was removed and centrifuged gently at 300 x g for 10 min to collect any cells that were dislodged due to cell death. This cell pellet was combined with the cells harvested after trypsinization for 3 min at 37°C. The cells were washed with PBS and pelleted at 1800 rpm for 10 min at RT, and used for staining for MBP and active caspase-3 as described below.

For flow cytometry staining of MBP, cells harvested from the various conditions were distributed into aliquots of cell suspensions adjusted to a cell count of 1 x 10^6^, each in a total volume of 250 μL of PBS, followed by fixation and permeabilized using 250 μL of Cytofix/Cytoperm (BD Biosciences, San Diego, CA, USA) for 20 min at RT in the dark with gentle rocking. Cells were then washed in 1 mL of Perm/Wash buffer (BD Biosciences) and pelleted at 700 x g for 10 min at RT. Cell pellets were resuspended in 150 μL of PBS and incubated with 20 μL of primary rabbit anti-MBP antibody (Millipore) for 60 min at RT. Stained cells were then washed once with the Perm/Wash buffer as described above, resuspended in 150 μL of PBS, and stained further with 1 μL (2 μg of protein) of secondary antibody, goat anti-rabbit IgG-Alexa 488 (Invitrogen) for 30 min at RT in the dark. Cells were then washed with the Perm/Wash buffer and fixed using 300 μL of 2% PFA.

For detection of oligodendrocyte apoptosis, cells were previously stained for MBP using primary and secondary antibody as described, and washed and pelleted using the Perm/Wash buffer. Cell pellets were then resuspended in 150 μL of PBS and incubated for 1 h at RT with 20 μL of phycoerythrin (PE)-conjugated anti-active caspase-3 antibody (BD), in the dark, for active caspase-3 staining. Respective controls were included for cells without antibodies, single-stain controls for primary MBP antibody, secondary antibody anti-rabbit Alexa 488, and PE-active-caspase-3 only, for compensation settings. Cells were then washed and pelleted as described above, and finally fixed using 300 μL of 2% PFA and kept protected from light at 4°C until analyzed. As no non-specific binding with isotype control for MBP was previously found in the immunofluorescence staining method described above, no isotype control was included here for flow cytometry evaluation.

Flow cytometric acquisition was performed within 24 h of staining. At least 100,000 events were collected from each sample using a FACS Calibur instrument (BD Biosciences). Data were analyzed using FlowJo software (TreeStar, Inc.) version 9.0.1.

### Statistical evaluation

The unpaired-two tailed t test was used to evaluate the statistical significance between means of datasets, using Graphpad Prizm software (Graph Pad Software Inc.) version 4.

## Results

### Expression of the mature oligodendrocyte marker MBP by differentiated MO3.13 cells and differentiated HOPC

**Figure 1:**
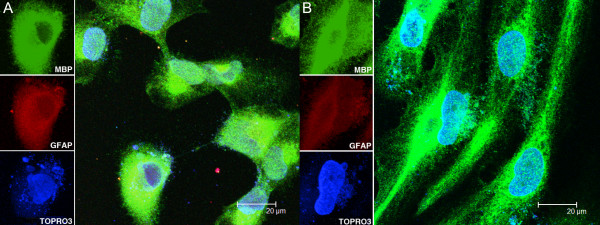
**Cell morphology, myelin basic protein, and glial fibrillary acidic protein expression in MO3.13 oligodendrocyte cultures.** MO3.13 cultures maintained in growth medium show expression of myelin basic protein (MBP, in green) and glial fibrillary acidic protein (GFAP red) (**A**). TOPRO3, a nuclear stain, appears blue. Upon differentiation, when growth medium is replaced with differentiation medium, cells change in morphology, continue to show expression of MBP, and the expression of GFAP is decreased (**B**).

MO3.13 cell cultures held in growth medium expressed both MBP and GFAP (Figure 1A). Upon differentiation, mature MO3.13 oligodendrocytes showed elongated cell processes and continued to express MBP, while showing reduced GFAP expression as compared to undifferentiated cells (Figure 1B). Differentiated HOPC also expressed MBP (not shown in Figure 1, but see Figure 5 B-D). Oligodendrocytes incubated with respective isotype controls and corresponding secondary antibodies did not show any detectable signal (not shown).

### Pro-inflammatory response induced by *B. burgdorferi* in MO3.13 oligodendrocytes

**Figure 2:**
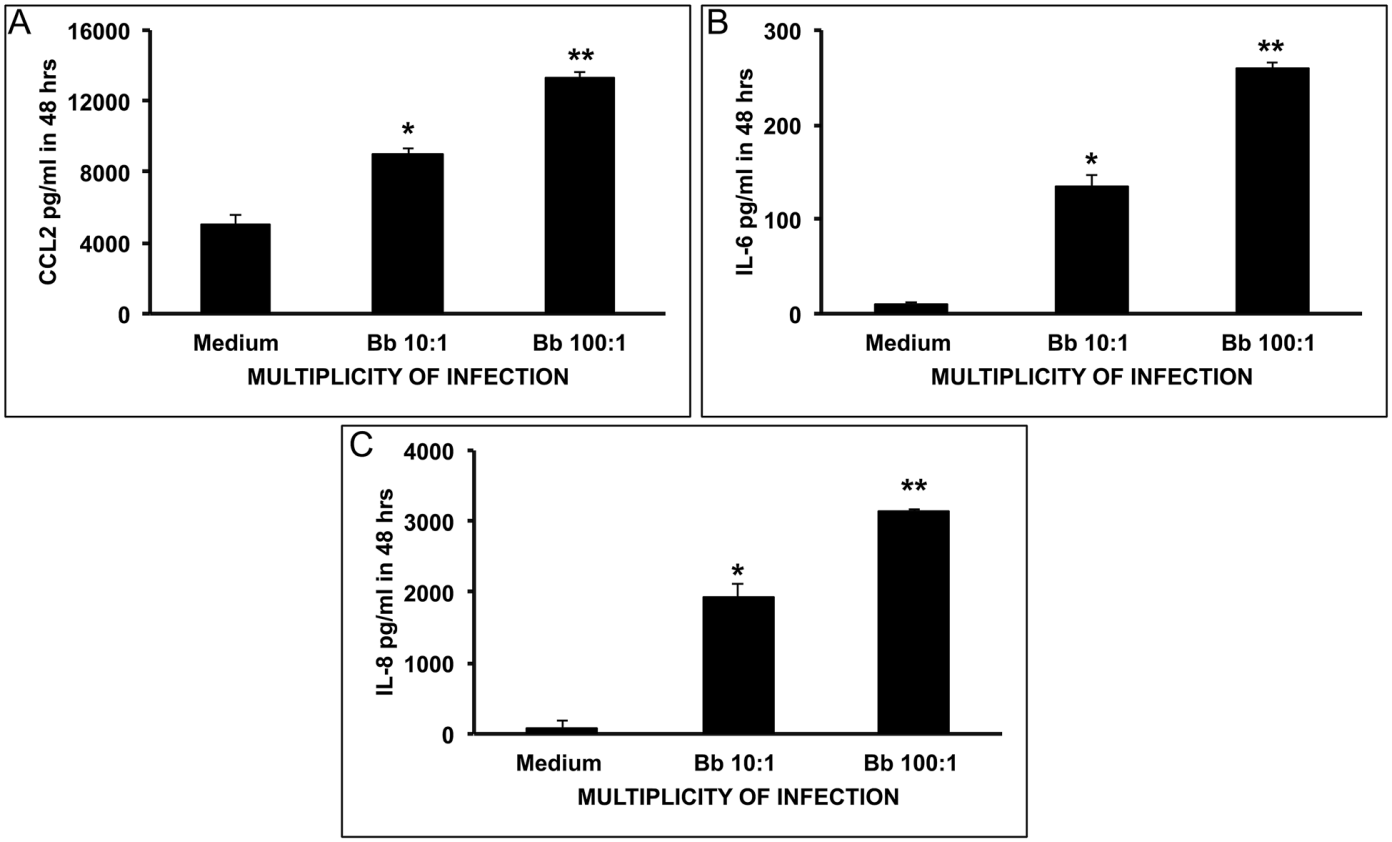
***B. burgdorferi*induces CCL2, IL-6 and IL-8 in MO3.13 oligodendrocytes in a dose dependent fashion.** Evaluation by the multiplex ELISA assay of culture supernatants of differentiated MO3.13 oligodendrocytes incubated with live *B. burgdorferi* spirochetes at a multiplicity of infection (MOI) of 10:1, and 100:1 for 48 h show elevated levels of CCL2 (**A**), IL-6 (**B**), and IL-8 (**C**) as compared to that observed in medium controls (* *P* < 0.05, ** *P* < 0.01).

Live *B. burgdorferi* spirochetes incubated with differentiated MO3.13 cell cultures for 48 h at a MOI of 10:1 and 100:1 induced significantly elevated levels of CCL2 (Figure 2A), IL-6 (Figure 2B) and IL-8 (Figure 2C) as compared to the levels induced in medium controls. The concentration of CCL2 surpassed 8,000 pg/mL and 13,000 pg/mL at MOI of 10:1 and 100:1, respectively, whereas the constitutive level of this chemokine that was produced in medium alone was of 5,000 pg/mL (Figure 2A). The basal concentration of IL-6 was of only approximately 10 pg/mL but reached more than 130 pg/mL and 250 pg/mL at MOI of 10:1 and 100:1, respectively (Figure 2B). IL-8 production displayed a similar pattern but with higher values than IL-6 (Figure 2C). *B. burgdorferi* also induced marginally higher levels of the cytokines GMCSF and IFN-γ in a dose-dependent manner as compared to controls (not shown). Data represent mean values and standard deviations between values of two independent experiments. The concentration values in each of the two experiments are the mean of duplicate determinations within the experiment.

### Evaluation of apoptosis of MO3.13 oligodendrocytes in the presence of *B. burgdorferi*

**Figure 3:**
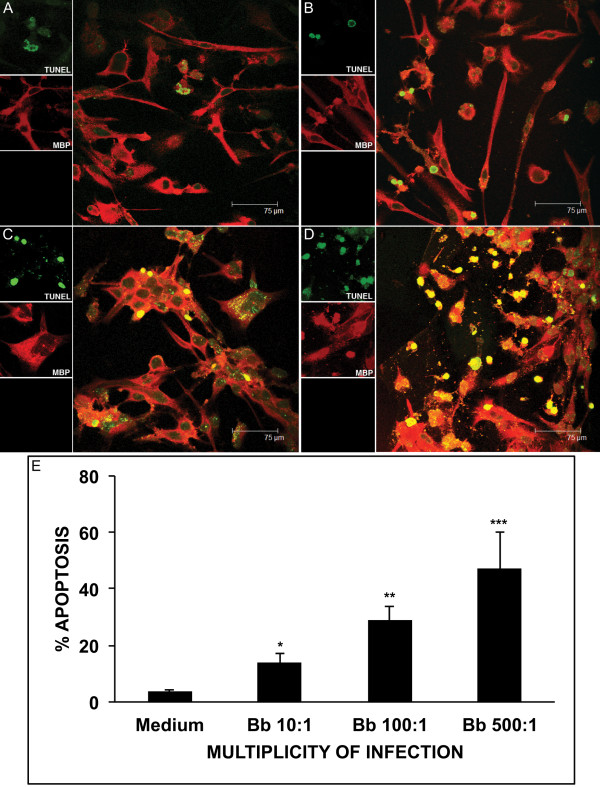
***B. burgdorferi*induces apoptosis in MO3.13 oligodendrocytes in a dose-dependent manner.** Apoptosis detected by the *in situ* TUNEL assay (green) in MBP-stained differentiated MO3.13 cells (red), in medium control (**A**) and after incubation with live *B. burgdorferi* for 48 h at multiplicity of infection (MOI) of 10:1 (**B**), 100:1 (**C**), and 500:1 (**D**), as visualized by confocal microscopy. (**E**) : Graphical representation of the percent apoptosis as detected by the *in situ* TUNEL assay in differentiated MO3.13 cells held in medium control and after incubation with live *B. burgdorferi* at MOIs of 10:1, 100:1, and 500:1, respectively, for 48 h (*P < 0.05, **P < 0.01, ***P < 0.001).

Live *B. burgdorferi* induced apoptosis, as detected by the *in situ* TUNEL assay, in differentiated MO3.13 oligodendrocytes, after 48 h of incubation. Apoptosis visualized by confocal microscopy in medium alone, and after incubation with live *B. burgdorferi* at MOI of 10:1, 100:1, and 500:1 are shown in Figures 3 (A-D), respectively. The mean percent apoptosis and standard deviations quantified from ten microscope fields (a total of 500 cells) for each condition is shown in Figure 3E.

### Effect of the anti-inflammatory drug dexamethasone on the pro-inflammatory response elicited by *B. burgdorferi* in differentiated MO3.13 oligodendrocytes and differentiated HOPC

**Figure 4:**
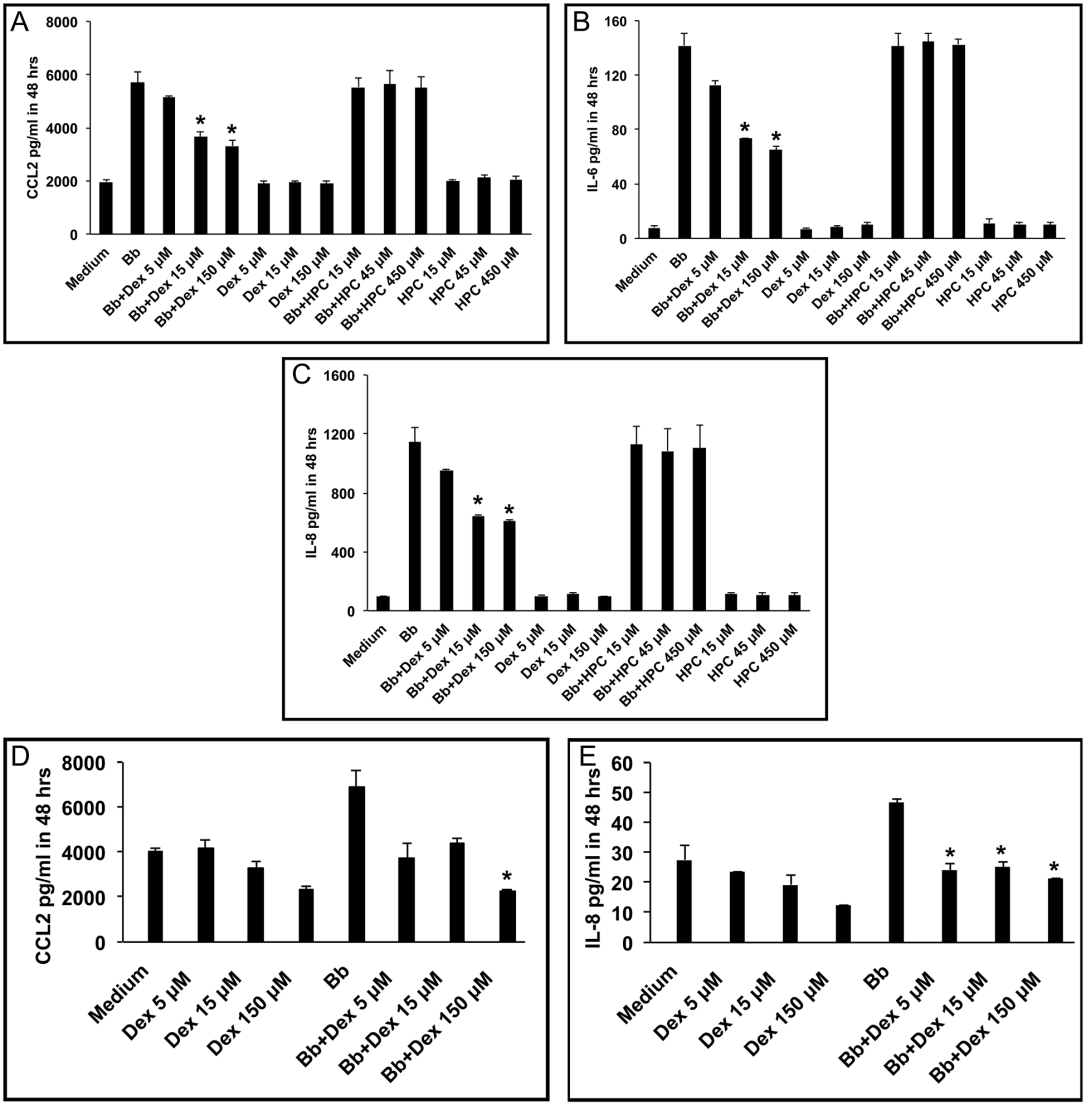
**Dexamethasone reduces levels of CCL2, IL-6, and IL-8 induced by*B. burgdorferi*in differentiated oligodendrocytes.** Evaluation by the multiplex ELISA assay of culture supernatants of differentiated MO3.13 oligodendrocytes incubated with live *B. burgdorferi* spirochetes at a multiplicity of infection (MOI) of 10:1 for 48 h in the presence and absence of dexamethasone (Dex) at 5 μM, 15 μM, and 150 μM concentrations and carrier substance (2-hydroxypropyl)-β-cyclodextrin (HPC), at 15 μM, 45 μM, and 450 μM, respectively, showing levels of CCL2 (**A**), IL-6 (**B**), and IL-8 (**C**). Levels of CCL2 (**D**) and IL-8 (**E**) detected in culture supernatants of differentiated primary human oligodendrocytes, as induced by live *B. burgdorferi* after 48 h of incubation in the presence and absence of dexamethasone at 5 μM, 15 μM, and 150 μM concentrations (*P < 0.05).

Dexamethasone reduced the levels of CCL2, IL-6, and IL-8 as induced by live *B. burgdorferi* (MOI of 10:1) in MO3.13 oligodendrocytes after 48 h, as shown in Figures 4A, 4B, and 4C, respectively, in a dose-dependent fashion. Dexamethasone was able to significantly inhibit the levels of CCL2, IL-6, and IL-8 as induced by *B. burgdorferi* when used at 15 μM and 150 μM (Figure 4A, B, and C). We confirmed that the anti-inflammatory effect of the dexamethasone formulation was due to its dexamethasone fraction and not due to the carrier substance (2-hydroxypropyl)-β-cyclodextrin (HPC), as HPC alone at 15 μM, 45 μM, and 450 μM, the concentrations at which it is present in the dexamethasone concentrations used above, failed to reduce the levels of *B. burgdorferi*-induced immune mediators (Figure 4A, B, and C, respectively). Similarly, dexamethasone (5 μM, 15 μM, and 150 μM) reduced the levels of CCL2 and IL-8 as induced by live *B. burgdorferi* (MOI of 10:1) in HOPC cells after 48 h of co-incubation (Figure 4D and E).

### Effect of dexamethasone on apoptosis induced by *B. burgdorferi* in differentiated MO3.13 oligodendrocytes and differentiated HOPC

**Figure 5:**
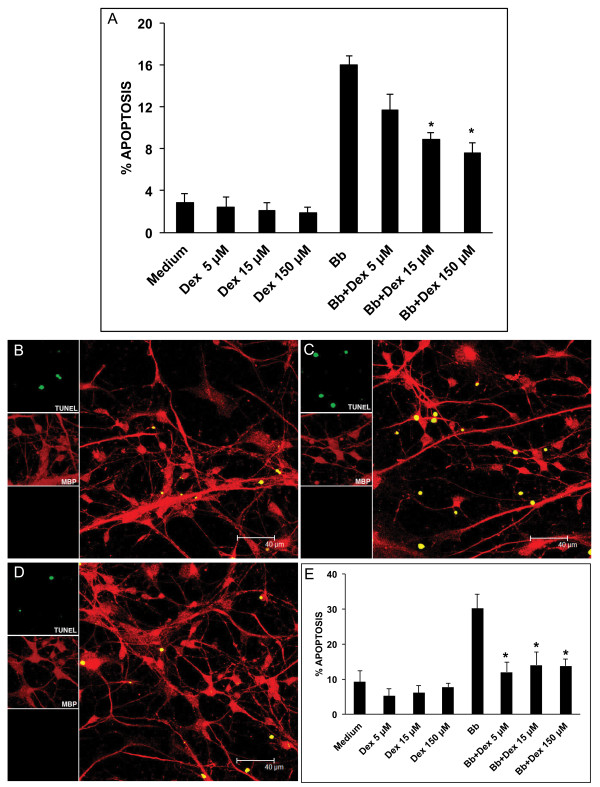
**Dexamethasone protects differentiated human oligodendrocytes from*B. burgdorferi*-induced apoptosis in a dose dependent fashion.** A graphical representation of the percent apoptosis as evaluated by the *in situ* TUNEL assay in differentiated MO3.13 oligodendrocytes (**A**) in the presence and absence of dexamethasone (Dex) at 5 μM, 15 μM, and 150 μM concentrations. Confocal images showing apoptosis as observed by the *in situ* TUNEL assay in differentiated human oligodendrocyte precursor cells (HOPC) incubated for 48 h in medium (**B**), *B. burgdorferi* at MOI of 10:1 (**C**) and *B. burgdorferi* (10:1) in the presence of dexamethasone at 5 μM (**D**). The TUNEL signal is seen in green in differentiated HOPC showing expression of myelin basic protein (MBP), in red. (**E**) Graphical representation of the protective effect of dexamethasone on *B. burgdorferi*-induced apoptosis in HOPC at 5 μM,15 μM, and 150 μM concentrations, (* *P* < 0.05).

Figure 5A shows percent apoptosis in MO3.13 oligodendrocytes as measured by the *in situ* TUNEL assay after 48 h of incubation in medium control (2.85% *±* 0.91), with *B. burgdorferi* alone (16% *±* 0.89) at MOI of 10:1, *B. burgdorferi* + dexamethasone as well as medium + dexamethasone at 5 μM, 15 μM, and 150 μM. Dexamethasone was protective against *B. burgdorferi*-induced apoptosis, showing significant reduction in apoptosis at 15 μM (8.93 *±* 0.65) and 150 μM (7.6 *±* 0.99) (*P* < 0.05). However, when dexamethasone was used at the high concentration of 1,500 μM it appeared to be toxic, as it not only resulted in higher levels of apoptosis than that induced by *B. burgdorferi* alone (84.4% *±* 1.2), but it also induced high levels of apoptosis in cells incubated in medium alone (75.8%) (not shown in Figure 5A).

Live *B. burgdorferi* also induced enhanced apoptosis as evaluated by the *in situ* TUNEL assay and visualized by confocal microscopy in differentiated HOPC cells, Figure 5C, as compared to that seen in medium controls, Figure 5B, after 48 h of incubation. A confocal image of the protective effect of dexamethasone at 5 μM on *B. burgdorferi*-induced apoptosis is shown in Figure 5D. Figure 5E shows a graph of the percent apoptosis observed in HOPC cells when incubated with live *B. burgdorferi,* and medium controls in the presence and absence of dexamethasone at 5 μM, 15 μM, and 150 μM after 48 h of incubation. Dexamethasone significantly reduced the levels of *B. burgdorferi*-induced apoptosis in HOPC cells from (30.19% *±* 4.12) to 12.05% *±* 2.8 in the presence of 5 μM dexamethasone, 14.05% *±* 3.58 at 15 μM, and 13.79% *±* 2 at 150 μM, respectively (*P* < 0.05).

### Caspase-3 activation induced by *B. burgdorferi* in differentiated MO3.13 oligodendrocytes

**Figure 6:**
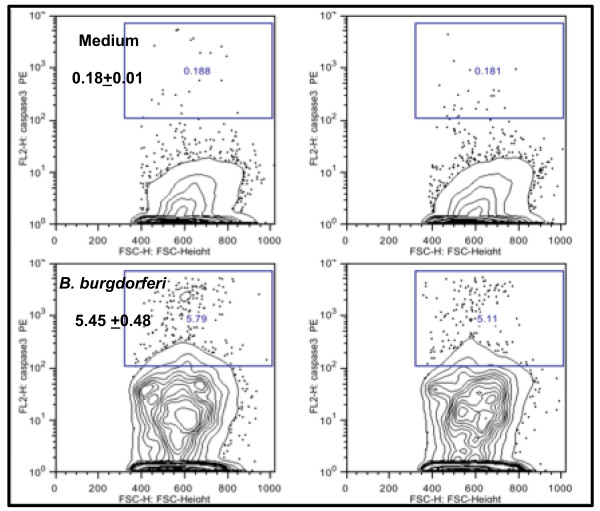
***B. burgdorferi*induces elevated levels of activated caspase-3 in differentiated MO3.13 cells.** Flow cytometric evaluation of activated caspase-3 in differentiated MO3.13 cells following incubation in differentiation medium (top panel) and with live *B. burgdorferi* (MOI, 10:1) for 48 h (bottom panel). The mean percent of MBP-positive cells showing positive staining for activated caspase-3 in cultures held in medium (0.18 *±* 0.1) was elevated by 30-fold in MO3.13 cells incubated with live *B. burgdorferi* (5.45 *±* 0.48). The figure shows the results of two independent experiments.

Caspase-3 activation was quantified by flow cytometry in differentiated MO3.13 cells in the presence and absence of live *B. burgdorferi* (Figure 6). Cells were incubated with spirochetes for 48 h. The percent of MBP-positive cells showing positive staining for activated caspase-3 in cultures held in medium (0.18 *±* 0.1) was elevated by 30-fold in MO3.13 cells incubated with live *B. burgdorferi* (5.45 *±* 0.48). The results represent the mean and standard deviation of values obtained from two independent experiments.

## Discussion

In recent studies, using *ex vivo* and *in vivo* modes of experimentation in the rhesus monkey model of LNB, we had established that *B. burgdorferi* is able to induce inflammatory mediators, with concomitant apoptosis of oligodendrocytes in the frontal cortex, and of satellite glial cells in dorsal root ganglia [39,40]. We had also shown with experiments performed *in vitro* that human neurons co-cultured with *B. burgdorferi* and rhesus microglia undergo apoptosis in the presence of pro-inflammatory mediators chiefly produced by the microglia [44]. In this study we focused on evaluating the ability of live *B. burgdorferi* to induce oligodendrocyte damage in an *in vitro* system, using differentiated MO3.13 human oligodendrocytes and differentiated HOPC. We addressed the hypothesis that inflammation plays a role in mediating apoptosis of oligodendrocytes, as induced by *B. burgdorferi*, using the anti-inflammatory drug dexamethasone. We included HOPC in our study to corroborate the observations that we made with the MO3.13 cell line.

We established *in vitro* cultures of MO3.13 cells and confirmed the presence of phenotypic markers that are known to be expressed by this cell line, namely MBP and GFAP [41].

Our first key observation was that *B. burgdorferi* is able to induce the pro-inflammatory mediators CCL2, IL-6, and IL-8 in oligodendrocytes. The levels of immune mediators detected in the culture supernatants increased concordantly with an increase in the spirochetal MOI. HOPC similarly produced CCL2 and IL-8, a finding that further validates the results obtained with MO3.13 cells. These observations echo our previous findings made with astrocytes and microglia, as these glial cells also produced pro-inflammatory mediators in response to live *B. burgdorferi*, and expand the scope of our hypothesis of a role for glial cells in mediating inflammation in LNB [39,40,44-46]. Oligodendrocytes could therefore contribute to the elevated levels of cytokines and chemokines detected in the CSF of patients with LNB [35-38].

Cytokines and chemokines play a central role in inflammation, demyelination, and neurodegeneration in the CNS during inflammatory neurodegenerative diseases such as multiple sclerosis (MS) [47]. Oligodendrocytes in brain tissue that is immediately adjacent to the subarachnoid space, the region known as the sub-pial space, are especially vulnerable to demyelination [48]. Since inflammatory lesions are commonly found in the meninges in LNB, the myelitis that is seen in LNB may be in part due to oligodendrocytes. These cells could be damaged by the inflammatory process brought about by the oligodendrocytes themselves, with participation of other glial cells, in addition to inflammatory mediators produced by the perivascular cellular infiltrates that are often present in CNS infection. Oligodendrocytes are known to express receptors for various cytokines and chemokines [49].

CCL2 was induced at high levels in oligodendrocytes by *B. burgdorferi*. This chemokine is of particular importance in mediating inflammation in neurodegenerative diseases [50]. CCL2 recruits monocytes and T cells from the blood stream into the CNS during acute neuroinflammation, in addition to recruiting microglia, the resident macrophages of the brain [51]. It is an important mediator in many neuroinflammatory and neurodegenerative brain diseases characterized by neuronal degeneration [52]. CCL2 has been found to be up-regulated in actively demyelinating MS plaques [53], and its expression is increased in experimental autoimmune encephalomyelitis [54]. It is known to modulate microglial activation and proliferation, thus contributing to the inflammatory response mounted by the CNS [55]. Importantly, CCL2 levels are elevated in the CSF of patients with LNB [56], and we found high levels of CCL2 in the CSF of rhesus monkeys infected intrathecally with *B. burgdorferi*[40]. CCL2 also has been documented to play a role in mediating nerve damage and demyelination of axons by causing influx of monocytes and T cells, in Wallerian degeneration [57,58], and may thus contribute to the axonal damage that affects patients with LNB of the PNS [10,11].

The cytokine IL-6, which was also elevated in the culture supernatants of oligodendrocytes that were exposed to live *B. burgdorferi,* is known to be both helpful and harmful in the CNS [31-34]. Dysregulated expression of IL-6 has been documented in several neurological disorders such as MS, acute transverse myelitis, Alzheimer’s disease, schizophrenia, epileptic seizures, and Parkinson’s disease [49]. In addition, IL-6 has been shown to be involved in multiple physiological CNS processes such as neuron homeostasis, astrogliogenesis, and neuronal differentiation [59]. Elevated levels of IL-6 have also been found in the CSF of LNB patients [35]. IL-6 is known to promote oligodendrocyte and neuronal survival in the presence of glutamate-mediated excitotoxicity in hyppocampal slices [60]. IL-6 is also known to support survival of oligodendrocytes *in vitro*[61].

The third pro-inflammatory mediator whose concentration was significantly increased in culture supernatants of oligodendrocytes stimulated with live *B. burgdorferi* is IL-8. This chemokine also has been reported to be elevated in the CSF of LNB patients [62]. We had previously documented that *B. burgdorferi* induces production of IL-8 in rhesus microglia, astrocytes and endothelial cells [39,40,44,46]. IL-8 released into the CSF after brain injury is associated with blood-brain barrier dysfunction and plays a central role in recruitment of neutrophils and T cells into the CNS during bacterial meningitis [63,64].

Our second key observation was that live *B. burgdorferi* induce a significantly elevated level of apoptosis, as assessed by the TUNEL assay, in MO3.13 oligodendrocytes compared to that seen in medium controls. The level of apoptosis observed increased concordantly with an increase in the *B. burgdorferi* MOI. We also observed elevated levels of activated caspase-3, a phenomenon that is known to be an early signaling event that results in apoptosis [65]. The MO3.13 oligodendrocyte cell line used in these studies has also been shown to undergo active caspase-3-mediated apoptosis due to other stimuli such as ceramide [66,67], and inflammatory cytokines [68]. Caspase-1, -2 and -3 are known to be expressed in mature oligodendrocytes [69]. Caspase-mediated oligodendrocyte cell death (particularly via activation of caspase-11 and caspase-3) has also been documented in inflammatory demyelinating diseases such as MS [70].

The interaction of *B. burgdorferi* with oligodendrocytes resulted in elevated levels of inflammatory mediators and concomitant apoptosis in oligodendrocytes, suggesting that the phenomena of inflammation and apoptosis might be causally related. To uncover the possible connection between inflammation and apoptosis in this system we treated both differentiated MO3.13 cells as well as differentiated HOPC with the anti-inflammatory drug dexamethasone. In both cases the effect was not only a reduction in the amount of pro-inflammatory mediators, as would be expected in the presence of dexamethasone, but also a significant reduction in the fraction of cells undergoing apoptosis. This outcome is a strong indication that inflammation plays a role in mediating oligodendrocyte apoptosis.

Cytokines such as TNF, IL-1β, lymphotoxin (LT), and TGF-β are known to cause cell death in oligodendrocytes [71-74]. TNF and IL-1β were not detected in the culture supernatants of oligodendrocytes that were incubated with live *B. burgdorferi* for 48 h*.* TGF-β and LT were not among the mediators that were detected by the human 14-plex array that we used and may well have been present in the culture supernatants. TNF, LT, [71] and TGF-β [72] were shown to induce apoptosis in oligodendrocytes when added exogenously, while IL-1β caused glutamate-mediated excitotoxic death of oligodendrocytes co-cultured with astrocytes and microglia [73], or when injected intra-cerebrally in neonatal rats [74].

The potential of CCL2, IL-6, and/or IL-8 to induce oligodendrocyte apoptosis has not been documented thus far in the literature. In fact, IL-6 is known to promote the survival of oligodendrocytes in culture [61]. IL-8 has been shown to induce the expression of pro-inflammatory proteases, matrix metalloproteinases MMP-2 and MMP-9, cell-cycle protein cyclin D1, an early marker for G1/S transition and pro-apoptotic protein Bim (Bcl-2-interacting mediator of cell death), and cell death in cultured neurons in 24 h [75]. CCL2 is implicated in mediating oligodendrocyte/white matter damage indirectly by mediating the influx of immune cells such as T cells and macrophages, resulting in cytotoxic damage of the myelin sheath of axons, followed by phagocytosis of myelin debris, culminating in demyelination and axonal damage [76]. A possible involvement of cytotoxic cells in the immune response against *B. burgdorferi* has been suggested based on *in vitro* studies [77], in addition to reports indicating the presence of a cytolytic phenotype of IFN-γ producing cells from patients with LNB [78]. It is likely that a similar mechanism may be mediating the demyelination and axonal degeneration resulting in white matter lesions seen in LNB [4,6,18-22].

The anti-inflammatory effect of dexamethasone, a glucocorticoid used in the treatment of immune-mediated inflammatory diseases is well documented [42]. Dexamethasone has been shown to effectively reduce the levels of IL-6, IL-1β, and TNF released from human monocytes stimulated with endotoxin to below background levels [79]. Dexamethasone reduced the levels of CCL2 in brain and retinal vascular endothelial cells that were activated with pro-inflammatory cytokines IL-1β, TNF, and IFN-γ [80]. The anti-inflammatory potential of dexamethasone to reduce CCL2 and IL-8 also has been reported in cultured rheumatoid synoviocytes [81]. Here we show that dexamethasone can reduce the levels of CCL2, IL-6, and IL-8 as induced by *B. burgdorferi* in differentiated human oligodendrocytes. Clinical improvement was seen in a severe case of neuroborreliosis showing encephalomyelitis with polyneuropathy, when treated with the classically recommended 2 to 4 weeks of anti-microbial agents in combination with steroids [82].

Dexamethasone has been shown to suppress CCL2 production via mitogen-activated protein kinase phosphatase-1 (MAPK-P1)-dependent inhibition of Jun N-terminal kinase and p38 MAPK in activated rat microglia [43]. MAPK cascades are signal transduction pathways that play important regulatory roles in the biosynthesis of pro-inflammatory cytokines such as IL-6, IL-8, and CCL2 [83]. MAKP-P1, a member of the Map Kinase Phosphatase family, is essential for the dephosphorylation/deactivation of MAPK p38 and JNK, thereby limiting pro-inflammatory cytokine biosynthesis in innate immune cells exposed to microbial components or infectious agents [84]. MAPK such as p38 and JNK may be involved in the signaling mechanisms underlying both inflammation and apoptosis [83,85]. Earlier we had documented the role of p38 MAPK, Erk1, and Erk 2 in mediating the production of IL-6 and TNF, as well as apoptosis, in rhesus astrocytes as induced by lipoproteins of *B. burgdorferi*[86]. MAPK signaling pathways may indeed be involved in regulating both inflammation and apoptosis as induced by *B. burgdorferi* in human oligodendrocytes, as well as in the modulatory effect of dexamethasone that we observed.

## Conclusions

In this study we have established that live *B. burgdorferi* are capable of eliciting inflammatory mediators, particularly IL-6, IL-8, and CCL2, in addition to inducing apoptosis in human oligodendrocyte cultures *in vitro,* by activating caspase-3. Oligodendrocytes are the myelinating cells of the CNS that myelinate neuronal axons, providing saltatory conduction of action potentials and proper function of the CNS [87]. The role of oligodendrocyte death in MS is well established [88]. Some of the earliest pathological changes in inflammatory lesions seen in MS are increases in oligodendrocyte apoptosis [89,90]. Based on the observations of this study we propose that neurologic injury in the CNS during an infection with the Lyme disease spirochete *B. burgdorferi* could be mediated in part by the direct action of the spirochetes on oligodendrocytes or via inflammation mediated by *B. burgdorferi* in oligodendrocytes. As oligodendrocytes are vital for the survival and optimum function of neurons [91], oligodendrocyte damage could contribute to neuronal dysfunction and death and result in the impairment of CNS functions that are seen in patients with LNB.

## Competing interests

The authors declare that they have no competing interests.

## Authors’ contributions

GR participated in the design of the experiments, conducted cell culture experiments, multiplex ELISA data analysis, confocal microscopy, preparation and staining of samples for flow cytometry, and drafted the manuscript. SB helped in cell culture, immunofluorescence, and multiplex ELISA data analysis. BP performed the flow cytometry experiments. MP conceived of the study, contributed to the design of the experiments, and to drafting and editing the manuscript. All authors have read and approved the final version of the manuscript.
